# Characterization of the chromatin accessibility in an Alzheimer’s disease (AD) mouse model

**DOI:** 10.1186/s13195-020-00598-2

**Published:** 2020-03-23

**Authors:** Yaqi Wang, Xiaomin Zhang, Qiao Song, Yuli Hou, Jing Liu, Yu Sun, Peichang Wang

**Affiliations:** 1 Clinical Laboratory of Xuanwu Hospital, Capital Medical University, Beijing, 100053 People’s Republic of China; 2 Department of Neurology, Xuanwu Hospital, Capital Medical University, Beijing, 100053 People’s Republic of China

**Keywords:** Alzheimer’s disease, ATAC-seq, Chromatin accessibility, Transcription factors, RNA-seq

## Abstract

**Background:**

The pathological hallmarks of Alzheimer’s disease (AD) involve alterations in the expression of numerous genes associated with transcriptional levels, which are determined by chromatin accessibility. Here, the landscape of chromatin accessibility was studied to understand the outline of the transcription and expression of AD-associated metabolism genes in an AD mouse model.

**Methods:**

The assay for transposase-accessible chromatin by sequencing (ATAC-seq) was used to investigate the AD-associated chromatin reshaping in the APPswe/PS1dE9 (APP/PS1) mouse model. ATAC-seq data in the hippocampus of 8-month-old APP/PS1 mice were generated, and the relationship between chromatin accessibility and gene expression was analyzed in combination with RNA sequencing. Gene ontology (GO) analysis was applied to elucidate biological processes and signaling pathways altered in APP/PS1 mice. Critical transcription factors were identified; alterations in chromatin accessibility were further confirmed using chromatin immunoprecipitation assays.

**Results:**

We identified 1690 increased AD-associated chromatin-accessible regions in the hippocampal tissues of APP/PS1 mice. These regions were enriched in genes related to diverse signaling pathways, including the PI3K-Akt, Hippo, TGF-β, and Jak-Stat signaling pathways, which play essential roles in regulating cell proliferation, apoptosis, and inflammatory responses. A total of 1003 decreased chromatin-accessible regions were considered to be related with declined AD-associated biological processes including cellular response to hyperoxia and insulin stimulus, synaptic transmission, and positive regulation of autophagy. In the APP/PS1 hippocampus, 1090 genes were found to be upregulated and 1081 downregulated. Interestingly, enhanced ATAC-seq signal was found in approximately 740 genes, with 43 exhibiting upregulated mRNA levels. Several genes involved in AD development were found to have a significantly increased expression in APP/PS1 mice compared to controls, including *Sele*, *Clec7a*, *Cst7*, and *Ccr6*. The signatures of numerous transcription factors, including Olig2, NeuroD1, TCF4, and NeuroG2, were found enriched in the AD-associated accessible chromatin regions. The transcription-activating marks of H3K4me3 and H3K27ac were also found increased in the promoters of these genes. These results indicate that the mechanism for the upregulation of genes could be attributed to the enrichment of open chromatin regions with transcription factors motifs and the histone marks H3K4me3 and H3K27ac.

**Conclusion:**

Our study reveals that alterations in chromatin accessibility may be an initial mechanism in AD pathogenesis.

**Supplementary information:**

**Supplementary information** accompanies this paper at 10.1186/s13195-020-00598-2.

## Background

Alzheimer’s disease (AD), an age-associated chronic progressive neurodegenerative disorder, is characterized by progressive loss of memory, cognitive impairment, and behavioral changes. It is the most common form of dementia. A global systematic analysis of AD from 1990 to 2016 that included 195 countries showed that the global number of individuals who lived with dementia was 43.8 million in 2016, showing a 117% increase compared to 1990 [1]. In 2016, dementia was the fifth largest cause of death globally, accounting for 4.4% of total mortality. Notably, 8.6% of deaths were observed in individuals aged more than 70 years, making dementia the second largest cause of death in this age group [2].

The pathological hallmarks of AD are characterized by β-amyloid (Aβ) deposition, neurofibrillary tangles induced by phosphorylation of tau protein, upregulation of inflammation, and neuronal apoptosis. All these processes involve alterations in the expression and regulation of numerous genes. The Apolipoprotein E (*APOE*) gene is a well-known genetic risk factor identified for AD. The association of the *APOE* ε4 allele with AD risk has been repeatedly demonstrated by different studies, whereas the ε2 allele has been found to be related to a protective effect [3, 4]. Rare mutations in *APP*, *PSEN1*, and *PSEN2* are discovered to be associated with autosomal dominant familial AD, which is found in about 70% of familial AD patients [5–7]. With the development of next-generation sequencing (NGS) over the past few years, multiple AD genes have been identified. Rare variants in *TREM2* are considered a major genetic risk factor for AD [8–10]. In addition, both *SORL1* and *ABCA7* are demonstrated to carry numerous loss-of-function variants leading to strong increases in AD risk [11–14]. Moreover, a study from the Alzheimer’s disease sequencing project, which encompassed more than 5000 cases and controls, reported two new candidate genes, *IGHG3* and *ZNF655* [15]. Expression levels of AD genes are important in AD etiology; however, information on how they are specifically regulated is still limited. Thus, exploring the regulatory elements of AD genes and their corresponding transcription factors (TFs) is critically important for elucidation of the disease process.

In recent years, it has been recognized that chromatin is a dynamic central regulator of transcription. The chromatin structure defines the scenario where interactions between TFs and their cognate regulatory regions take place. To successfully interact with *cis*-regulatory elements, such as promoters, enhancers, insulators, and non-coding RNAs (ncRNAs), TFs must induce chromatin remodeling of nucleosomal structures, which results in different levels of chromatin accessibility [16, 17]. Therefore, open chromatin regions allow transcription machinery components to access to *cis*-regulatory elements and activate gene transcription, while closed chromatin regions impair the accessibility of promoters and enhancers to transcription factors and other regulators of transcription inducing gene silencing.

Recently, the results of several studies have demonstrated substantial changes in chromatin accessibility in human brains [18–20]. McClymont et al. reported the alteration of open chromatin in mouse dopamine neurons and found Parkinson-associated SNCA enhancer variants, indicating the utility of chromatin accessibility in studying the regulation of gene expression in neurodegenerative diseases [21]. However, the role of chromatin remodeling in AD processes has not been investigated yet.

In this work, we used the Assay for Transposase-Accessible Chromatin by sequencing (ATAC-seq), a sensitive tool for integrative epigenomic analysis, combined with RNA sequencing (RNA-seq) to investigate the pattern of genome-wide chromatin accessibility in AD in an APP/PS1 mouse model, which resembles the familial AD in humans, and characterize the chromatin accessibility landscape in APP/PS1 mice. We analyzed the differences in chromatin-accessible regions, which allowed us to identify the landscape of binding events, regulatory DNA sequences, and putative TFs that are likely responsible for these alterations.

## Methods

### Animal model

Male APPswe/PS1dE9 mice, 8 months of age (*n* = 3), and male, age-matched C57BL/6 mice (*n* = 3) were purchased from Nanjing Biomedical Research Institute of Nanjing University (Nanjing, China). All animals were housed in a specific pathogen-free room under a 12-h light-dark cycle with ad libitum access to food and water. Mice were acclimatized for a week, after which they were euthanized by cervical dislocation, and hippocampal tissues were collected. All animal procedures were performed according to the criteria outlined in the Guide for the Care and Use of Laboratory Animals (National Institutes of Health, Bethesda, MD) and with approval of the Animal Care and Use Committee of Xuanwu Hospital of Capital Medical University, China.

### Nuclei isolation, transposition reaction, and ATAC-seq library preparation

Nuclei were isolated from frozen hippocampal tissues. In brief, frozen hippocampal tissue (50–60 mg) was homogenized with a mortar and pestle on ice. The homogenized samples were placed in 1.0 mL of chilled lysis solution, mixed gently, and incubated on ice for 20 min. The mixture was then filtered through a cell-strainer (BD Biosciences, Franklin Lakes, NJ), mixed with 1.8 mL 1.8 M sucrose buffer, and loaded on the surface of a 1.0 mL 1.8 M sucrose cushion buffer. The nuclei were centrifuged at 27,550×g at 4 °C for 45 min and the pellets were resuspended in 0.5 mL chilled nuclei storage buffer and centrifuged at 500*g* at 4 °C for 5 min. The quality of the prepared nuclei was assessed with a light microscope. After the nuclei were counted, 50,000 nuclei were prepared for transposition reactions. The transposition reactions and ATAC-seq library preparation generally followed the steps set out in the original ATAC-seq paper using Nextera DNA Library Preparation Kit (Illumina, San Diego, CA) [22]. After transposition, DNA was purified with the MinElute PCR Purification Kit (Qiagen, Valencia, CA) and eluted in 10 μL elution buffer. The amplified libraries were purified with Ampure XP beads (Beckman Coulter) and were quantified with the Qubit dsDNA High Sensitivity Assay (Invitrogen, Carlsbad, CA) in combination with the High Sensitivity DNA Assay (Agilent, Santa Clara, CA) on the Agilent 2100 Bioanalyzer.

### ATAC-seq sequencing, library quality control, and data analysis

Each ATAC-seq library was sequenced on the Illumina Hiseq PE150 sequencer to obtain 80–100 million of 2 × 150 bp paired-end reads per sample. The quality of sequencing data was evaluated with FastQC (v.0.11.7, http://www.bioinformatics.bbsrc.ac.uk/projects/fastqc) and reads with Phred quality score > 30 were used for further analysis. Removing and trimming the adaptor sequences were performed to obtain clean data. The clean reads were then aligned to mouse genome mm10 with Burrows-Wheeler Aligner (0.7.10) [23]. Picard Tools (v.2.2.4, http://broadinstitute.github.io/picard) was used for duplicate removal. Samtools (v.1.3.1) was performed to filter multiply mapped reads, and BED tools were used for filtering out mitochondrial reads [24, 25]. Aligned reads from APP/PS1 mouse hippocampal tissues (8 months old, *n* = 3) or normal hippocampal samples from aged-matched control mice (*n* = 3) were merged and peak calling was conducted using MACS2 (v.2.1) [26]. Peaks that overlapped blacklisted regions were removed. The resulting sets of ATAC-seq peaks were considered as high confidence Tn5 hypersensitive site (THSS) regions. The annotation of THSS regions to genomic features was performed using the HOMER suite tool annotatePeaks (v.3.12) [27]. For the ACAT-seq enrichment analysis, we employed BEDTools (v.2.25.0) to obtain the count of reads overlapping with known transcription start sites (TSSs), promoters, exons, introns, etc. We considered the translation start sites as the reference point and − 1 Kb upstream of the translation start codon ATG as the putative promoter region. Differential accessibility analysis was conducted using DEseq2 (v.1.4.5) [28]. This analysis takes all the ATAC-seq peaks called in APP/PS1 mice and control mice and detects for normalized read count differences at the peak region. Motif discovery at chromatin-accessible regions was performed using the default setting in the HOMER suite function findMotifsGenome.pl tool [27]. Tracks were extracted and visualized using the UCSC genome browser [29]. Heatmaps showing ATAC-seq enrichment at promoters were built using the R statistical package [30].

### RNA-seq profiling and differential gene expression analysis

Total RNA was extracted using an RNeasy mini extraction kit (Qiagen), and RNA integrity was quantified by the Agilent RNA 6000 Nano Kit (Agilent) using the Agilent Bioanalyzer. Purified RNAs were reverse transcribed, and the resulting cDNA was further tagmented and PCR-amplified using the Nextera XT DNA sample kit (Illumina) to add the sequencing adaptors. The libraries were pooled and sequenced on the Illumina Hiseq PE150 sequencer to an average depth of 70 million reads per sample. The quality control of sequencing was performed via FastQC (v.0.11.7, http://www.bioinformatics.bbsrc.ac.uk/projects/fastqc). Reads were aligned to mm10 using the STAR splice-aware aligner (v.2.5.2b). QoRTs (v.1.0.7) were used to gather read counts per sample per gene for differential gene expression analysis [31]. Normalized RPKM (reads per kilo base million reads) values were used as a measure of gene expression. Differential gene expression analysis was performed using DESeq2 (5% FDR). The heatmap of RNA expression was produced using the correlation of normalized gene-level FPKM (fragments per kilobase million reads) values across samples with the heatmap.

### Chromatin accessibility and gene expression correlation

For purposes of assessing the global relationship between the ATAC-seq signal at promoters and the transcription levels of the corresponding genes, genes were categorized into high, medium, and low groups, based on their mRNA levels according to the RNA-seq data. A threshold value was determined by dividing the mRNA values (RPKM normalized values) in three quantile groups according to their means using the R statistical package. Violin plots were produced using ‘ggplot2’ from the R package. For comparison and visualization, ATAC-seq and RNA-seq enrichment data at various genomic features are shown on a log2 scale. In addition, the 25 most highly expressed genes and the 25 least highly expressed genes in APP/PS1 mice and controls were identified, and their transcriptional start sites (Ensembl) were extracted from the UCSC Table Browser. Regions expanded to 100 kb surrounding these TSSs were intersected and overlapped with the ATAC-seq libraries, then quantified and plotted, and the ATAC-seq signal over the most and least highly expressed genes was also quantified and plotted. In addition, the top 50 altered (highest and lowest) ATAC-seq peaks were extracted by *q* value, and the expression of the nearest gene was quantified and plotted as a final metric to relate the RNA-seq and ATAC-seq datasets.

### Gene ontology analysis

Gene ontology (GO) analysis was performed to facilitate elucidating the biological implications of unique genes in the significant profiles of the gene. We annotated the genes associated with the accessible chromatin regions using the Genomic Regions Enrichment of Annotations Tool (GREAT, v.3.0.0) [32]. The annotated genes were then analyzed based on the GO annotations in the database to obtain all the GO involved genes. The significant level of each GO was calculated by the Fisher test. *P* values < 0.05 were considered significant.

### Pathway analysis

Pathway analysis was performed to explore the annotated genes or differential genes enriched signaling pathways. The annotated genes or differential genes were analyzed based on KEGG database to obtain all the involved pathway terms. The significant level of each pathway term was calculated by the Fisher test. *P* values < 0.05 were considered significant.

### Chromatin immunoprecipitation and qPCR

Chromatin immunoprecipitation (ChIP) assay was performed using a Pierce™ Agarose ChIP Kit (Thermo Fisher, Rockford, IL) according to the manufacturer’s protocol. The hippocampal tissue lysates were incubated with anti-histone 3 trimethylated lysine 4 (ab8580, Abcam, Cambridge, UK), anti-histone 3 acetylated lysine 27 (ab4729, Abcam), or anti-rabbit IgG (ab171870, Abcam). Purified DNA and input DNA were analyzed by qPCR. The primers used for ChIP-qPCR are listed in Supplementary Table S1.

### Statistical analyses

Data are expressed as mean ± SD. Comparison between two groups was conducted with Student’s *t* test. Differences between groups were considered to be statistically significant at *P* < 0.05. Statistical analyses were performed using the GraphPad Prism 6 software (GraphPad, La Jolla, CA).

## Results

### Chromatin accessibility by ATAC-seq is predictive of active transcription in APP/PS1 mice

**Fig. 1:**
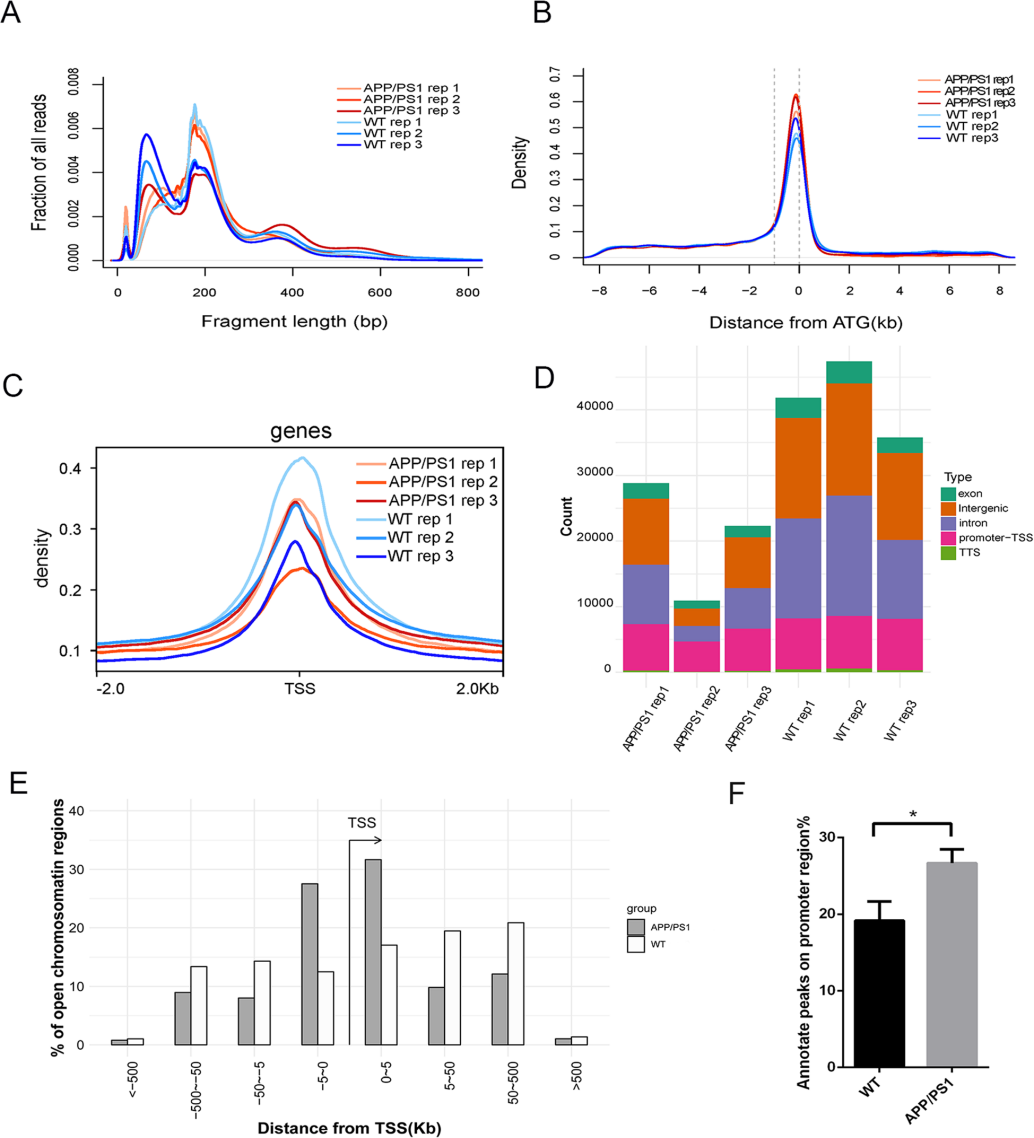
ATAC-seq chromatin accessibility analysis in hippocampus of Alzheimer’s disease (AD) model mice and wild type (WT) mice. **a** Distribution of ATAC-seq fragment size in AD and WT mice. **b** Density plot showing the position of THSSs in AD and WT mice. The left dashed line indicates the putative promoter region located 1 kb upstream. **c** Chromatin accessibility around the TSS in AD and WT mice. **d** Annotation of THSSs to genomic features: Exons, intergenic regions, introns, promoters, and TTS. THSSs located up to 1 kb upstream of the ATG are determined as promoter regions. **e** Representative distribution of chromatin-accessible regions across the genome in AD and WT mice. **f** The percentage of annotate peaks on promoter region in AD and WT mice. Data are shown as mean ± SD. *p < 0.05

As APP/PS1 mouse is a widely used animal model to study Alzheimer’s disease [33], we employed hippocampal tissues of APP/PS1 mice and age-matched wild type (WT) mice to generate ATAC-seq libraries. For each group, we obtained three independent ATAC-seq replicates. The similarity among replicates is high compared to the similarity between groups (Supplementary Figure S1). The chromatin was fragmented by Tn5 transposase into nucleosome-free, mono-nucleosome, and di-nucleosome patterns, and the similar distribution of fragment sizes suggested that chromatin is accessible to Tn5 transposase to the same degree in all samples independently between different groups (Fig. 1a). Distance analysis of ATAC-seq data showed that the location of THSS regions was mainly focused in a window of 1 kb upstream from the translation start codon (ATG) and was scarce at distances greater than 2 kb of the nearest gene (Fig. 1b). In addition, we found the ATAC-seq signal is significantly enriched at − 1Kb~+ 1Kb from TSSs (Fig. 1c). Based on the above findings, we annotated THSSs as promoters if the peak is located less than 1 kb upstream from the corresponding ATG (Fig. 1d) and used this annotation for the following analysis. The genomic location distribution of peaks showed that the peaks distributed on the promoter-TSS regions of APP/PS1 mice were significantly more than those of WT mice (26.66 ± 1.050%  vs 19.14 ± 1.449%), revealing that the accessibility of chromatin around the TSS regions of APP/PS1 mice was higher than that of WT mice (Fig. 1e, f).

**Fig. 2:**
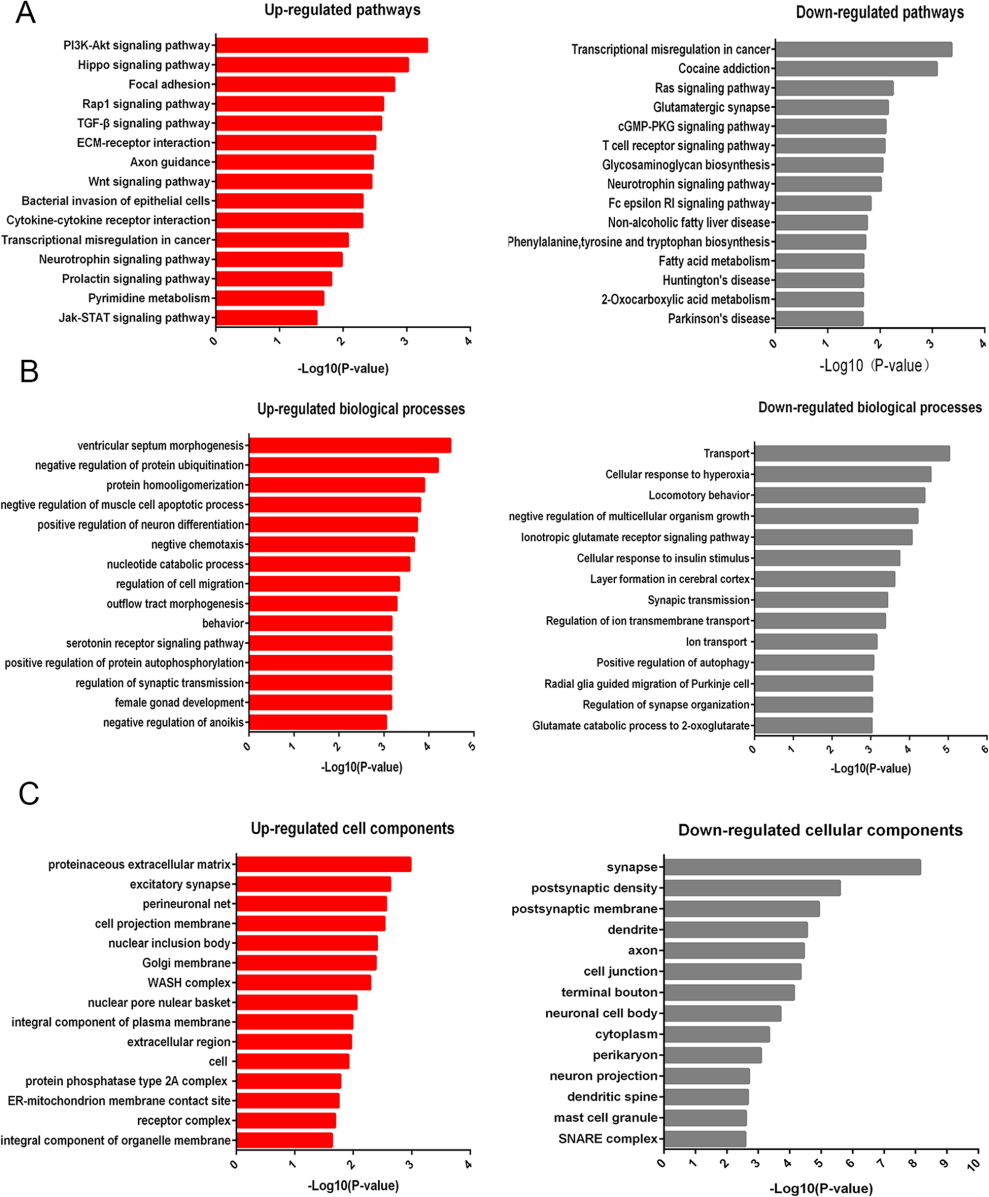
Pathway analysis and Gene Ontology (GO) analysis in AD mice. **a** Signaling pathway associated with chromatin accessibility in AD mice. **b** GO analysis of biological process associated with accessible chromatin regions in AD mice. **c** GO analysis of cellular component associated with chromatin-accessible regions in AD mice

AD-associated metabolism involves numerous genes that participate in multiple signaling pathways. To investigate the functional role of the chromatin-accessible regions in the regulation of gene expression involved in signaling pathways and gene function, we analyzed different pathways and biological processes in AD model mice and WT mice. Our results showed that 204 annotated genes were associated with chromatin-accessible regions in AD mice and were involved in active signaling pathways. These regions were enriched in different signaling pathways including the PI3K-Akt, Hippo, TGF-β, and Jak-Stat signaling pathway that play essential roles in regulating of cell proliferation, cell apoptosis, and inflammatory responses (Fig. 2a, Supplementary Table S2). In consideration that neuronal death and microglia proliferation increase in AD mice, they may account for some of changes of signaling pathway above in AD [34–38]. Meanwhile, 237 annotated genes were found to associate with chromatin accessibility in decreased signaling pathways including RAS signaling pathway, glutamatergic synapse, and glycosaminoglycan biosynthesis (Fig. 2a, Supplementary Table S3). GO analysis showed that several AD-associated biological processes were reduced including cellular response to hyperoxia and insulin stimulus, synaptic transmission, and positive regulation of autophagy (Fig. 2b, Supplementary Table S4), while ventricular septum morphogenesis, negative regulation of protein ubiquitination, and protein homooligomerization were induced in AD-associated biological processes (Fig. 2b, Supplementary Table S5). In addition, some critical cellular components were deficient in the hippocampus of AD mice, such as synapse, postsynaptic membrane, dendrite, axon, neuronal cell body, etc (Fig. 2c, Supplementary Table S6), and the increased cell components were also identified (Fig. 2c, Supplementary Table S7).

### Differential chromatin accessibility in APP/PS1 mice associated with gene expression

**Fig. 3:**
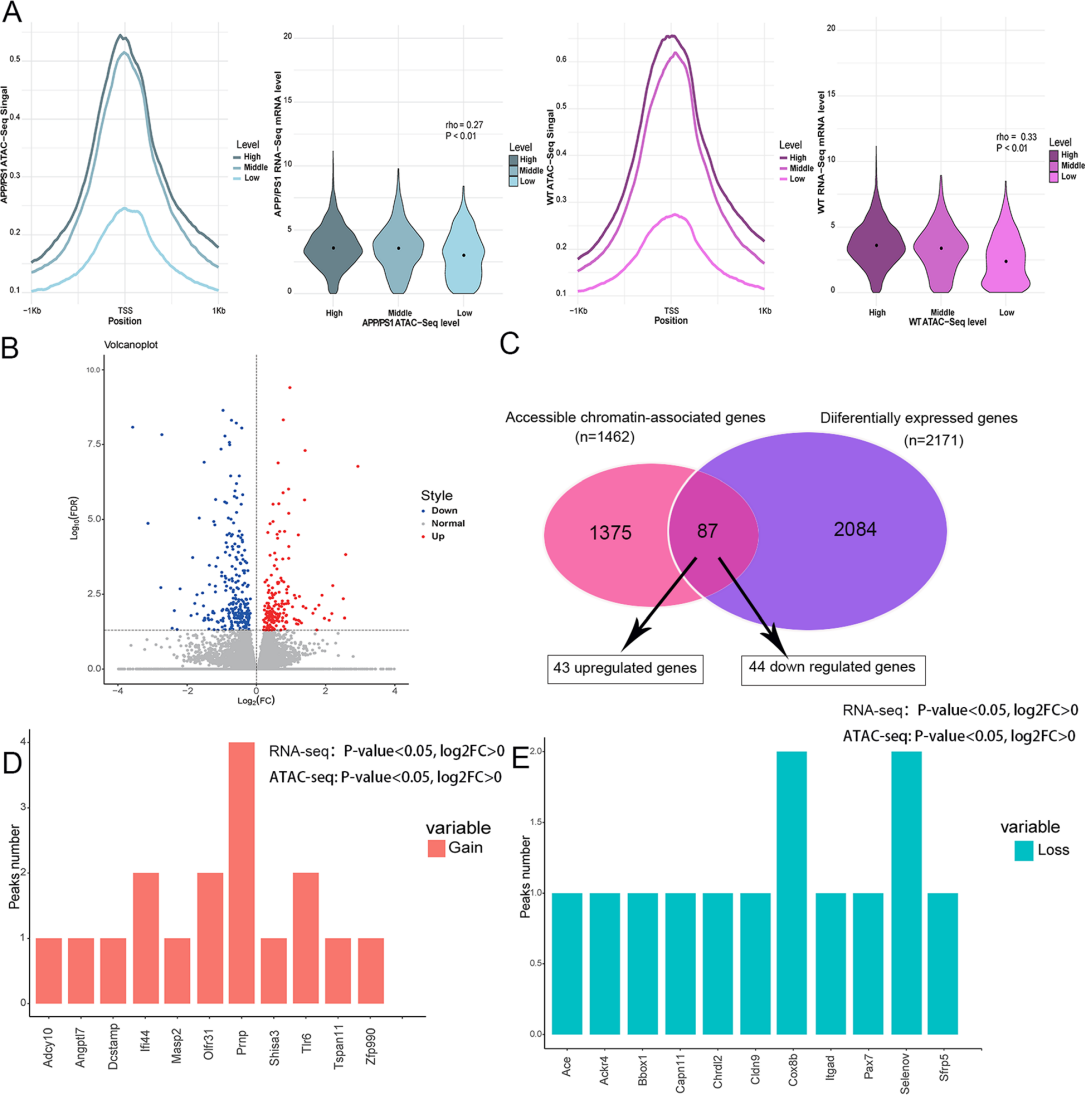
The association between the AD-specific chromatin-accessible regions and gene expression in AD mice. **a** ATAC-seq signal at TSSs correlates quantitatively with gene expression, the left figures (in blue) show the correlation between ATAC-seq signal at TSS and gene expression in AD mice, and the right figures (in pink) show the correlation between ATAC-seq signal at TSS and gene expression in WT mice. **b** Volcano plot of differentially expressed genes in AD, upregulated genes are shown by red dots and downregulated genes are shown by blue dots. **c** Venn diagram showing genes associated with the chromatin-accessible regions in AD and differentially expressed genes. **d** Upregulated genes in AD that are associated with AD-specific open chromatin regions. **e** Downregulated genes in AD that are associated with AD-specific closed chromatin regions

To explore the relationship between chromatin accessibility and transcription genome-wide, we performed RNA-seq and integrated with ATAC-seq data that computed the ATAC-seq signal level at known TSSs in groups of genes categorized with low, medium, and high mRNA level. Results revealed that the level of enrichment in ATAC-seq signal at the TSS region positively correlated with mRNA abundance of the annotated gene (Fig. 3a), indicating that highly transcribed genes showed a more open chromatin landscape than genes with low transcriptional level. Moreover, we found that the gene expression differed significantly between AD model mice and WT mice. We examined 1690 increased AD-associated chromatin-accessible regions and 1003 decreased AD-associated chromatin-accessible regions from ATAC-seq data. Meanwhile, a total of 1090 upregulated and 1081 downregulated genes were identified in AD model mice from RNA-seq data (Fig. 3b). In addition, we detected 740 genes associated with an increased ATAC-seq signal, of which 43 exhibited upregulated mRNA levels, and 722 were associated with decreased ATAC-seq signal, of these, 44 genes displayed downregulated mRNA levels (Fig. 3c, Supplementary Table S8, Supplementary Table S9). Furthermore, the region around high expression genes in the AD hippocampus, such as *Prnp*, *Olfr31*, and *Ifi44*, had gained open chromatin architecture, whereas regions around low expression genes, for example *Cox8b*, *Selenov*, and *Pax7*, showed lost chromatin signals (Fig. 3d, e).

**Fig. 4:**
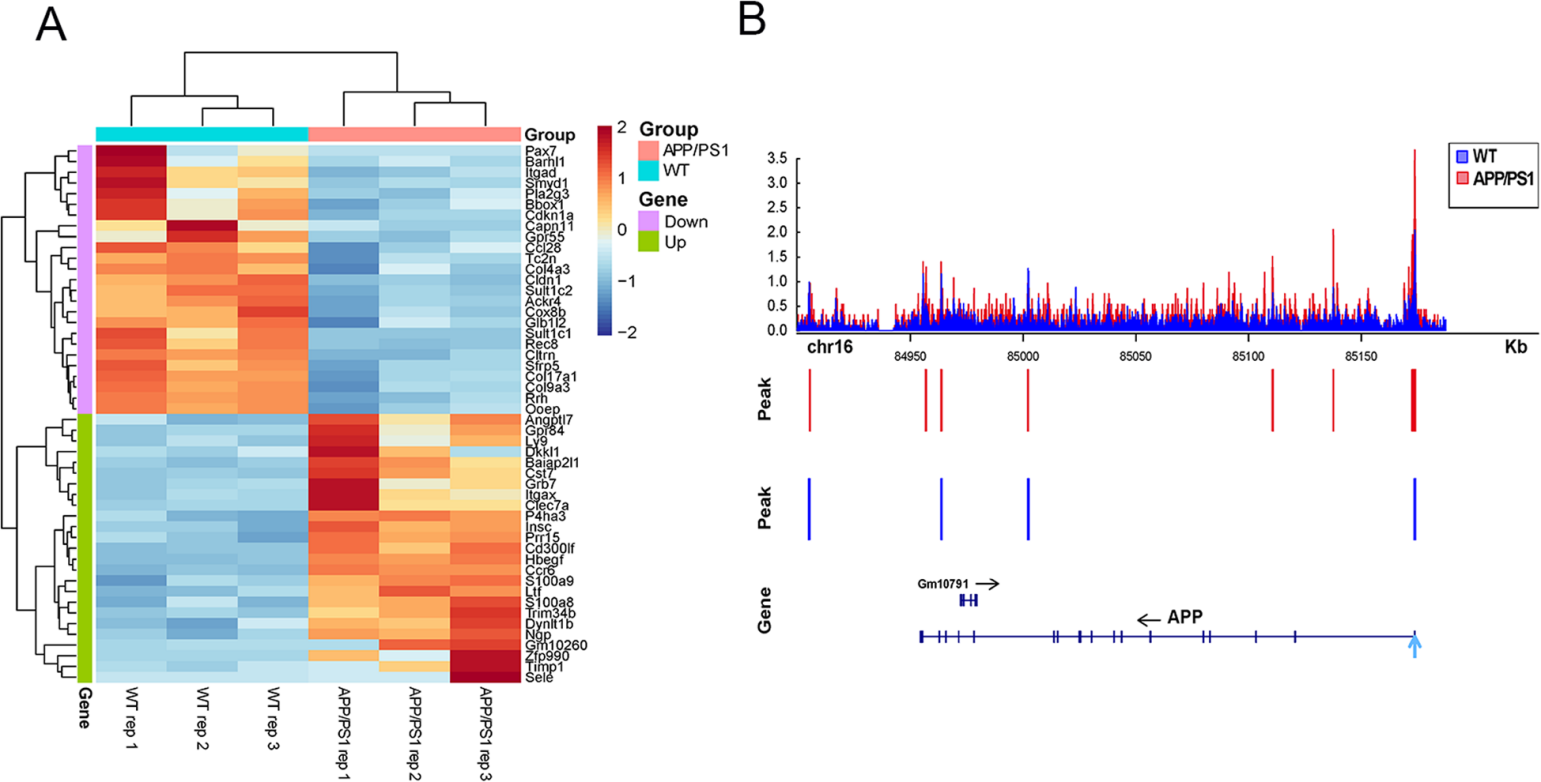
RNA-seq gene expression analysis in the hippocampus of AD and WT mice. **a** Heatmap of 50 most variable genes are listed based on RNA-seq. **b** Changes in chromatin accessibility downstream of the *APP* gene. Track in blue shows normalized and input-corrected ATAC-seq signal in WT mice and track in red shows normalized and input-corrected ATAC-seq signal in APP/PS1 mice. The chromatin-accessible regions are indicated with blue bars (WT mice) or red bars (AD mice) on the middle area of the graph and the TSS is shown by blue arrow

**Fig. 5:**
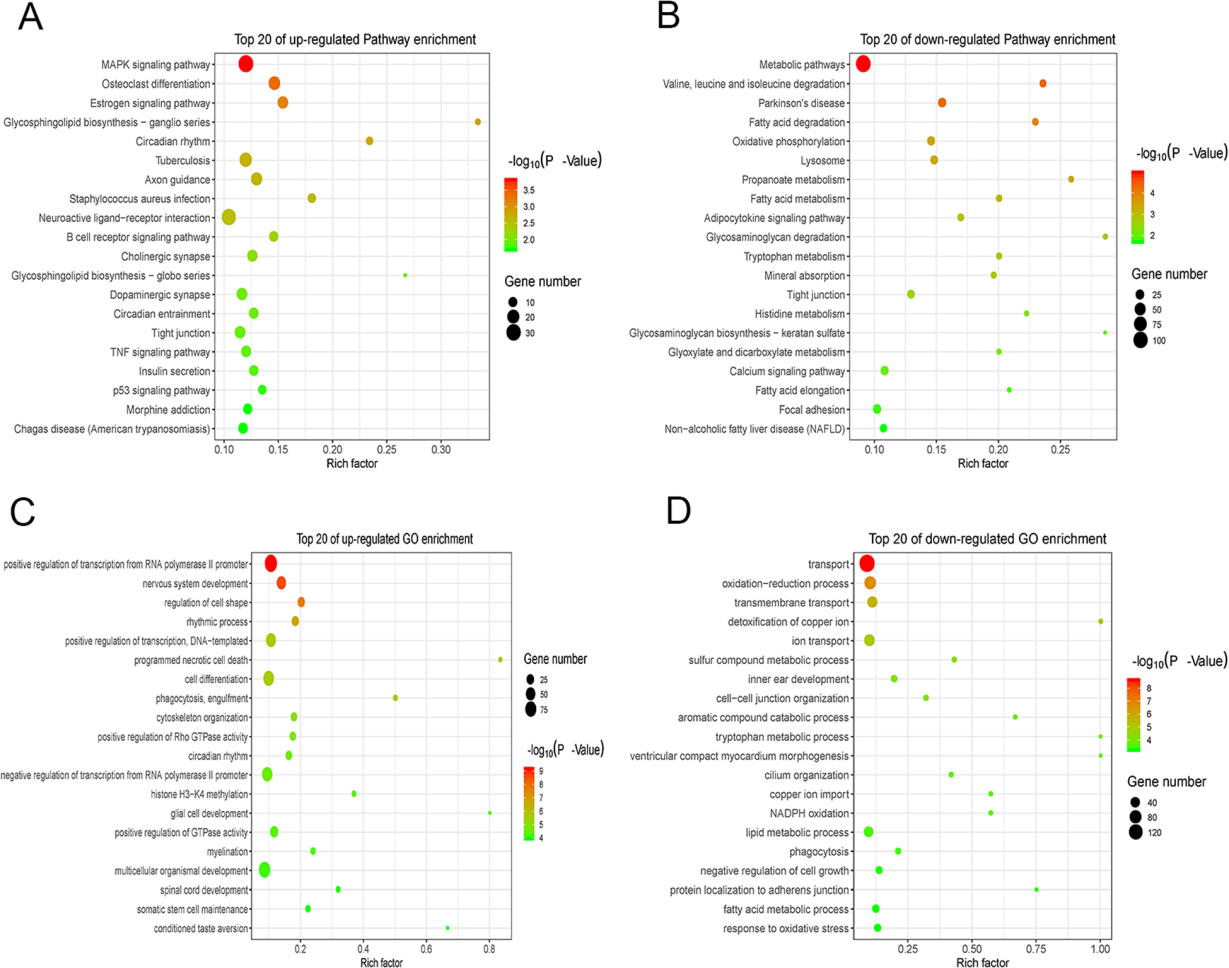
Gene enrichment in signaling pathways and GO analysis. **a** Upregulated gene enrichment in signaling pathways (20 most enrichment signaling pathways are listed). **b** Downregulated gene enrichment in signaling pathways (20 most enrichment signaling pathways are listed). **c** Upregulated gene enrichment in biological process (20 most enrichment biological process are listed). **d** Downregulated gene enrichment in biological process (20 most enrichment biological process are listed). The rich factor is defined as the ratio of the number of differential genes enriched in the pathway to the number of annotated genes

The heatmap of RNA-seq is shown in Fig. 4a, and the 50 most variable genes in AD mice are listed. The *APP* gene, which encodes the amyloid protein precursor, was also found upregulated in AD mice (Supplementary Table S10). Furthermore, we observed the chromatin accessibility of *cis*-regulatory elements in the *APP* gene. The results showed that the locus containing the upstream *APP* enhancer exhibited higher chromatin accessibility (Fig. 4b). These results indicate that the AD-associated open chromatin regions play a functional role in AD-specific transcriptome aberrations. In addition, the upregulated differential genes expressed in AD mice were enriched in the MAPK signaling pathway, osteoclast differentiation, estrogen signaling pathway, and neuroactive ligand-receptor interaction (Fig. 5a). Meanwhile, the downregulated differential genes expressed in AD mice were enriched in metabolic pathways, valine, leucine, and isoleucine degradation and fatty acid degradation (Fig. 5b). Moreover, the GO analysis showed that the upregulated genes in AD were enriched in nervous system development, histone H3-K4 methylation, and glial cell development (Fig. 5c), and downregulated genes in AD were enriched in oxidation-reduction process, ion transport, and lipid metabolic process (Fig. 5d).

### Chromatin accessibility footprints identify critical transcription factors in AD mice

**Fig. 6:**
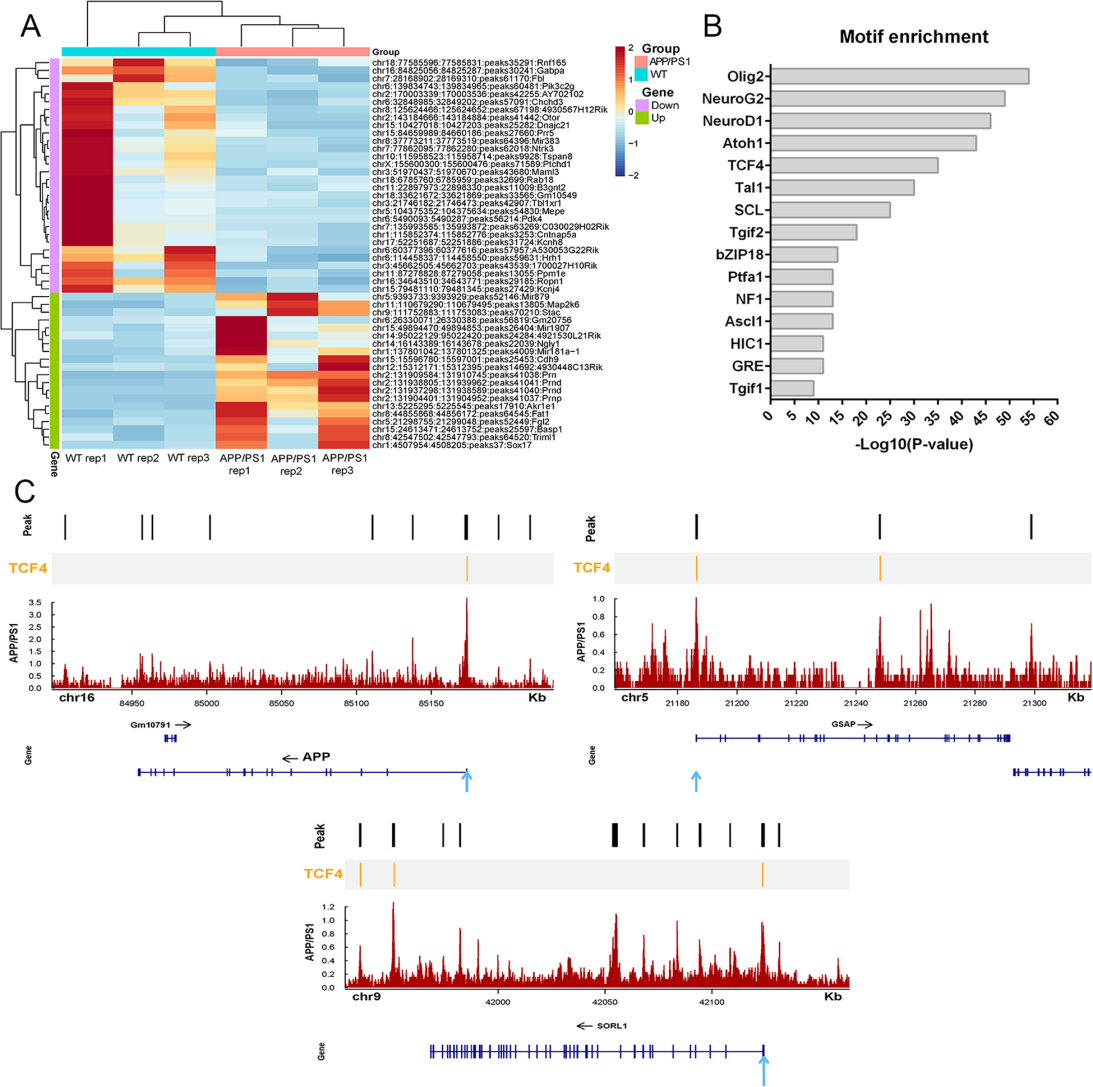
Motif enrichment at chromatin-accessible regions in AD mice. **a** Heatmap of 50 most enrichment ATAC-seq peaks at accessible chromatin regions. **b** The 15 motifs with the greatest enrichment. **c** Tracks for AD sample at the *APP*, *GSAP*, and *SORL1* genes with predicted TCF4 binding sites. The predicted TCF4 binding sites are shown with orange bars on the upper areas of the tracks. The TSS is indicated by blue arrow

To elucidate potential mechanisms of the relationship between over-expression genes and open chromatin regions, we investigated DNA transcription factor binding at AD-associated chromatin-accessible sites. The 50 most differential peaks of ATAC-seq in AD model mice and WT mice are shown in Fig. 6a. Moreover, we identified 15 most enriched transcriptional factor motifs in the increased chromatin-accessible regions in AD (Fig. 6b). Among the top 15 enriched motifs, those of Olig2, NeuroG2, NeuroD1, Atoh1, and TCF4 have been demonstrated to play key roles in AD. Furthermore, the TCF4 motif was identified in the open chromatin region around the TSS in AD-specific genes such as *APP*, *GSAP*, and *SORL1* (Fig. 6c). In addition, motif enrichment for regions of decreased chromatin accessibility in AD is showed in Supplementary Figure S2.

### Histone H3K4me3 and H3K27ac marks are enriched in open chromatin regions in AD model mice

**Fig. 7:**
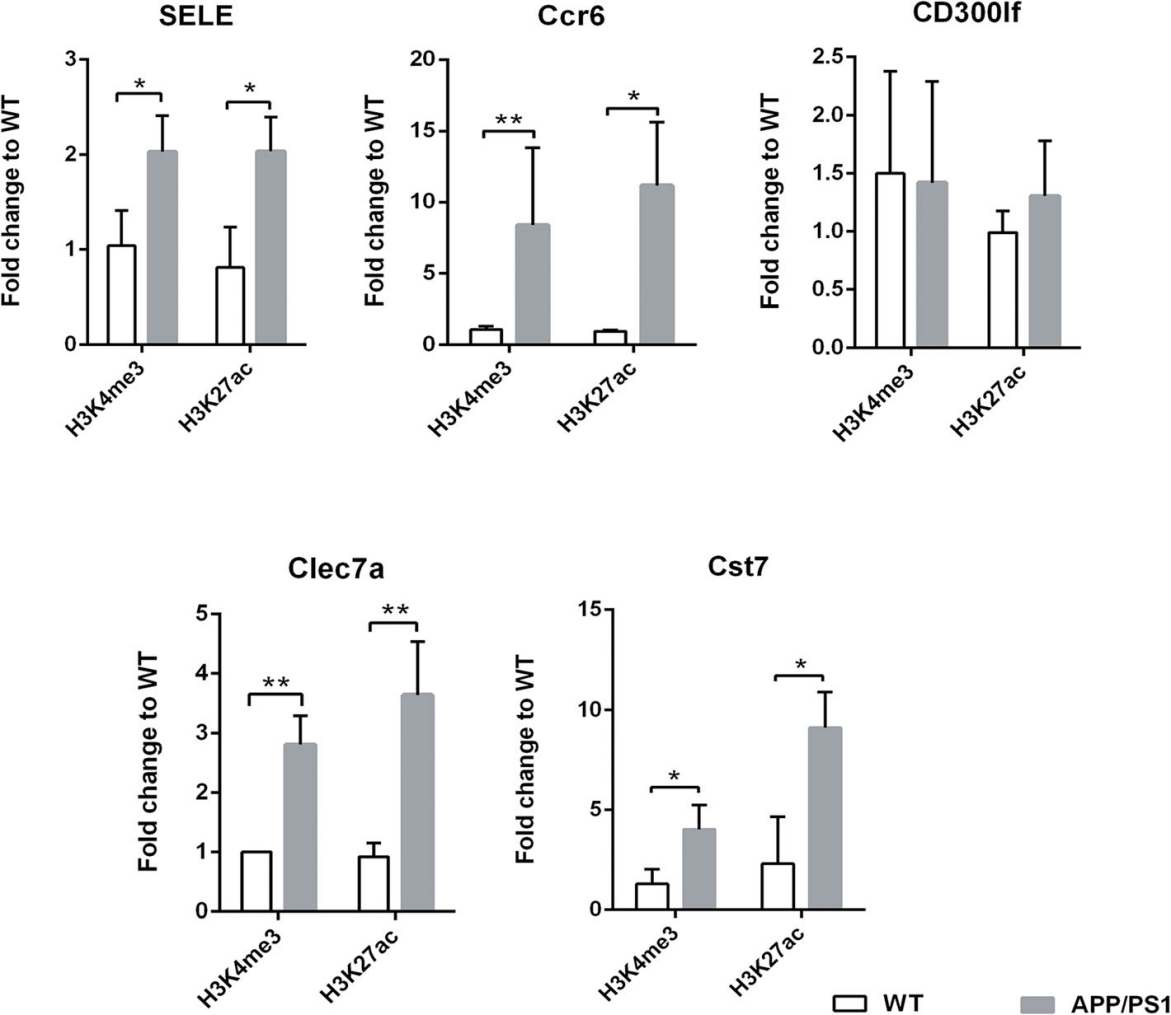
Histone modifications at the accessible chromatin regions. The fold changes of histone H3K4me3 and H3K27ac are determined by ChIP-qPCR, in chromatin-accessible regions of *SELE*, *Ccr6*, *CD300lf*, *Clec7a*, and *Cst7* genes. Data are shown as mean ± SD. *p < 0.05 and **p < 0.01

It is well established that active histones are important marks for gene regulation, as H3K4me3 and H3K27ac are characteristic of TSS/promoter region and are associated with TF binding [39–41]. Thus, we investigated the histone modifications in open chromatin regions of the most upregulated genes in AD mice, including *Sele*, *Ccr6*, *CD300lf*, *Clec7a*, and *Cst7*. The results showed that four of the five most upregulated genes, except for *CD300lf*, displayed a higher level for both H3K4me3 and H3K27ac marks in chromatin-accessible gene regions in the hippocampus of AD model mice than in WT mice. Notably, the level of H3K27ac in the *Cst7* gene and the levels of H3K4me3 and H3K27ac in the *Ccr6* gene exhibited more than fivefold increases (Fig. 7). These results indicate that H3K4me3 and H3K27ac marks correlating positively with chromatin accessibility regulate gene expression.

## Discussion

The genome-wide landscape of AD has been studied widely, and the gene expression patterns involved in in the process of AD have been described in detail [42, 43]. However, the mechanism of functional genome alterations in AD requires further investigation. Recent advances in chromatin profiling, especially genome-wide chromatin accessibility profiling methodologies, including MNase-seq, DNase-seq, FAIRE-seq, and ATAC-seq, have made it possible to analyze the alterations in the accessible chromatin state and allow us to better understand the molecular mechanisms of numerous diseases such as cancer, malaria, and metabolic disorders [44–47]. Although DNase-seq and MNase-seq can provide some subsets of the information obtained by ATAC-seq, they still have some limitations, for example large cell numbers, longer experimental time, and limited applicability to many systems. ATAC-seq offers substantial advantages over existing technologies due to its speed, simplicity, and low input cell number requirement [48]. In the present study, we performed ATAC-seq to examine the chromatin accessibility in AD model mice, demonstrated significant alterations in chromatin accessibility, and described transcriptional profiles in AD hippocampal samples.

Our results showed that the most accessible regions localize around the TSS and that open chromatin at these sites is predictive of active transcription, in agreement with the pattern reported in previous studies [46]. Given that recent large-scale association studies suggested genes related to AD risk are involved in many different biological pathways [42], we analyzed different biological processes and pathways in AD model mice and WT controls. Among the signaling pathways, we found to be strongly associated with AD were the Hippo and TGF-β, known to induce cell apoptosis [49, 50]. Regarding Hippo signaling, a recent study reported that the precursor of Aβ can promote the nuclear translocation of Foxo3a by inducing MST1-dependent phosphorylation of Foxo3a. The MST-Foxo pathway is considered a branch of the Hippo pathway; it activates a proapoptotic member of the Bcl-2 family triggering an intrinsic apoptotic pathway, thus resulting in neuronal death [51]. Relevant to TGF-β signaling, different studies have shown increased expression of TGF-β1 and TGF-β2 in brains of individuals with AD [52–54]. Given the extensive evidence of microglial dysfunction in neurodegeneration, including changes in microgial phagocytic activity, it is possible that changes in brain TGF-β signaling in AD could alter microglial state and trigger their pathogenic functions [55].

We also found the Jak-Stat and PI3K/Akt signaling pathways to strongly associate with AD. Several studies have demonstrated the Jak-Stat signaling as a possible underlying pathogenetic mechanism of AD, showing the importance of inflammatory pathways involved in AD [56, 57]. Besides, the PI3K/Akt pathway has been found to be related to tau protein hyper-phosphorylation [58]. The alterations of the chromatin accessibility in these signaling pathways may partly elucidate the mechanism of pathological changes in AD such as neurofibrillary tangles induced by phosphorylation of tau protein, the upregulation of inflammation, and neuronal apoptosis.

To observe the differential chromatin accessibility in APP/PS1 mice associated with gene expression, we examined 740 chromatin-accessible regions in AD with 43 upregulated associated genes and identified several genes with a notable increasing expression, including *Sele*, *Clec7a*, *Cst7*, *Ccr6*, and *CD300lf*, which may have profound significance in the AD process. The *Sele* gene encodes a cell-surface glycoprotein E-selectin that has a role in immunoadhesion [59]. It is thought to be responsible for the accumulation of leukocytes at sites of inflammation by mediating the adhesion of cells to the vascular lining. Li et al. reported that E-selectin in CSF was significantly elevated in clinically diagnosed AD patients without the typical AD CSF biomarker signature compared to those with a positive biomarker signature [60]. Clec7a and Cst7 play key roles in immune regulation. Recent studies have demonstrated that Clec7a and Cst7 are upregulated in the switch from homeostatic microglia to disease-associated microglia, which is found in AD at the proximity of the Aβ plaques [61, 62]. Additionally, highly significant increases of Ccr6 expression were observed in the brain and spleen of both the younger and older 3 × Tg-AD mice, implicating Ccr6 may be a possible biomarker for the development of AD-like disease [63]. On the other hand, we also identified enriched motifs at chromatin-accessible regions in AD, for example motifs for Olig2, NeuroG2, NeuroD1, Atoh1, and TCF4. Several of the most upregulated genes and AD-specific genes, we identified possess the enriched motif binding sites around the chromatin-accessible regions of TSS, indicating that the motifs enriched in open chromatin regions may play important roles in regulating the gene expression that participates in the AD process.

Accessibility at regulatory regions modulated by chromatin remodeling processes involves not only nucleosome occupancy, but also histone modifications, especially H3K4me3 and H3K27ac, which are necessary for transcription factor binding. Therefore, we investigated these histone modifications in the upregulated genes in AD. We found that both H3K4me3 and H3K27ac marks are highly expressed in the promoter regions of *Sele*, *Clec7a*, *Cst7*, and *Ccr6* genes. A recent study has reported the patterns of H3K27ac in entorhinal cortex samples from AD cases and identified several H3K27ac-associated transcriptional variation genes, suggesting that histone marks may indeed be critical events causing activation of genes favoring the progression in AD [64].

## Conclusion

In summary, our work provides a novel strategy to study transcriptional regulation in AD through the description of open chromatin profiling by ATAC-seq. The utilization of this technique in AD allows us to identify the regulatory elements that play critical roles in the AD process. Furthermore, our data present an association between TF binding, chromatin-accessible regions, and gene regulation, providing new insights into the molecular mechanisms of AD. However, the future challenge is to elucidate how the alteration of TF influences the malfunctioning of critical genes involved in AD pathogenesis.

## Supplementary information

Additional file 1: **Supplementary Table S1.** List of primers used in ChIP-qPCR.

**Additional file 2.**

**Additional file 3.**

**Additional file 4.**

**Additional file 5.**

**Additional file 6.**

**Additional file 7.**

**Additional file 8.**

**Additional file 9.**

**Additional file 10.**

**Additional file 11.**

Additional file 12: **Figure S1.** Principal Component Analysis of ATAC-seq libraries in APP/PS1 mice and WT mice. The plot shows the percentage of variance explained by PC1 and PC2 axes and the distribution of the samples along the two first Principal Components.

Additional file 13: **Figure S2.** Motif enrichment for regions of decreased chromatin accessibility in AD.

## Abbreviations

ABCA7: ATP binding cassette subfamily A member 7
Aβ: β-amyloid
AD: Alzheimer’s disease
APP: Amyloid-beta precursor protein
ATAC-seq: The Assay for Transposase-Accessible Chromatin by sequencing
Atoh1: Atonal homolog 1
Bcl-2: B cell lymphoma-2
Ccr6: C–C motif chemokine receptor 6
Cd300lf: CD300 molecule like family member F
Clec7a: C-type lectin domain containing 7A
Cox8b: Cytochrome C oxidase subunit 8b
Cst7: Cystatin 7
Foxo3a: Forkhead box O3a
FPKM: Fragments per kilobase million reads
GO: Gene ontology
GSAP: Gamma-secretase-activating protein
H3K4me3: Histone 3 trimethylated lysine 4
H3K27ac: Histone 3 acetylated lysine 27
Ifi44: Interferon-induced protein 44
IGHG3: Immunoglobulin heavy constant gamma 3
Jak: Janus Kinase
NeuroD1: Neurogenic differentiation factor 1
NeuroG2: Neurogenin-2
NGS: Next-generation sequencing
Olfr31: Olfactory receptor
Olig2: Oligodendrocyte transcription factor 2
Pax7: Paired box protein 7
PI3K: Phosphatidylinositol 3 -kinase
PSEN1: Presenilin-1
PSEN2: Presenilin-2
Prnp: Prion protein
RPKM: Reads per kilobase million reads
Sele: E-selectin
Selenov: Selenoprotein V
SORL1: Sortilin related receptor 1
Stat: Signal transducer and activator of transcription
TCF4: Transcription factor 4
TGF-β: Transforming growth factor-β
THSS: Tn5 hypersensitive site
TSS: Transcription start site
TREM2: Triggering receptor expressed on myeloid cells 2
ZNF655: Zinc finger protein 655

## References

[1] GBD 2016 Dementia Collaborators (2019). Global, regional, and national burden of Alzheimer’s disease and other dementias, 1990–2016: a systematic analysis for the Global Burden of Disease Study 2016. Lancet Neurol.

[2] GBD 2016 Causes of Death Collaborators (2017). Global, regional, and national age-sex specific mortality for 264 causes of death, 1980–2016: a systematic analysis for the Global Burden of Disease Study 2016. Lancet.

[3] Stozicka Z, Zilka N, Novak M (2007). Risk and protective factors for sporadic Alzheimer’s disease. Acta Virol.

[4] Kim J, Basak JM, Holtzman DM (2009). The role of apolipoprotein E in Alzheimer’s disease. Neuron..

[5] Cruchaga C, Haller G, Chakraverty S, Mayo K, Vallania FL, Mitra RD (2012). Rare variants in APP, PSEN1 and PSEN2 increase risk for AD in late-onset Alzheimer’s disease families. PLoS One.

[6] Nicolas G, Wallon D, Jonsson T, Johannsdottir H, Ingason A, Helgason H (2016). Screening of dementia genes by whole-exome sequencing in early-onset Alzheimer disease: input and lessons. Eur J Hum Genet.

[7] Lanoiselee HM, Nicolas G, Wallon D, Rovelet-Lecrux A, Lacour M, Rousseau S (2017). APP, PSEN1, and PSEN2 mutations in early-onset Alzheimer disease: a genetic screening study of familial and sporadic cases. PLoS Med.

[8] Guerreiro R, Wojtas A, Bras J, Carrasquillo M, Rogaeva E, Majounie E (2013). TREM2 variants in Alzheimer’s disease. N Engl J Med.

[9] Jonsson T, Stefansson H, Steinberg S, Jonsdottir I, Jonsson PV, Snaedal J (2013). Variant of TREM2 associated with the risk of Alzheimer’s disease. N Engl J Med.

[10] Jin SC, Benitez BA, Karch CM, Cooper B, Skorupa T, Carrell D (2014). Coding variants in TREM2 increase risk for Alzheimer’s disease. Hum Mol Genet.

[11] Lambert JC, Ibrahim-Verbaas CA, Harold D, Naj AC, Sims R, Bellenguez C (2013). Meta-analysis of 74,046 individuals identifies 11 new susceptibility loci for Alzheimer’s disease. Nat Genet.

[12] Bellenguez C, Charbonnier C, Grenier-Boley B, Quenez O, LeGuennec K, Nicolas G (2017). Contribution to Alzheimer’s disease risk of rare variants in TREM2, SORL1, and ABCA7 in 1779 cases and 1273 controls. Neurobiol Aging.

[13] Pottier C, Hannequin D, Coutant S, Rovelet-Lecrux A, Wallon D, Rousseau S (2012). High frequency of potentially pathogenic SORL1 mutations in autosomal dominant early-onset Alzheimer’s disease. Mol Psychiatry.

[14] Steinberg S, Stefansson H, Jonsson T, Johannsdottir H, Ingason A, Helgason H (2015). Loss-of-function variants in ABCA7 confer risk of Alzheimer’s disease. Nat Genet.

[15] Bis JC, Jian X, Kunkle BW, Chen Y, Hamilton-Nelson KL, Bush WS, et al. Whole exome sequencing study identifies novel rare and common Alzheimer’s-Associated variants involved in immune response and transcriptional regulation. Mol Psychiatry. 2018;14 10.1038/s41380-018-0112-7. 10.1038/s41380-018-0112-7 PMC6375806 30108311

[16] Li B, Carey M, Workman JL (2007). The role of chromatin during transcription. Cell..

[17] Voss TC, Hager GL (2014). Dynamic regulation of transcription states by chromatin and transcription factors. Nat Rev Genet.

[18] de la Torre-Ubieta L, Stein JL, Won H, Opland CK, Liang D, Lu D (2018). The dynamic landscape of open chromatin during human cortical Neurogenensis. Cell..

[19] Fullard JF, Hauberg ME, Bendl J, Egervari G, Cirnaru MD, Reach SM (2018). An atlas of chromatin accessibility in the adult human brain. Genome Res.

[20] Bryois J, Garrett ME, Song L, Safi A, Giusti-Rodriguez P, Johnson GD (2018). Evaluation of chromatin accessibility in prefrontal cortex of individuals with schizophrenia. Nat Commun.

[21] Parkinson-Associated SNCA (2018). Enhancer variants revealed by open chromatin in mouse dopamine neurons. Am J Hum Genet.

[22] Buenrostro JD, Wu B, Chang HY, Greenleaf WJ (2015). ATAC-seq: a method for assaying chromatin Accessiblity genome-wide. Curr Protoc Mol Biol.

[23] Li H, Durbin R (2009). Fast and accurate short read alignment with Burrows-Wheeler transform. Bioinformatics..

[24] Li H, Handsaker B, Wysoker A, Fennell T, Ruan J, Homer N (2009). The sequence alignment/map format and SAMtools. Bioinformatics..

[25] Quinlan AR (2014). BEDTools: the Swiss-Army tool for genome feature analysis. Curr Protoc Bioinformatics.

[26] Zhang Y, Liu T, Meyer CA, Eeckhoute J, Johnson DS, Bernstein BE (2008). Model-based analysis of ChIP-Seq (MACS). Genome Biol.

[27] Heinz S, Benner C, Spann N, Bertolino E, Lin YC, Laslo P (2010). Simple combinations of lineage-determining transcription factors prime cis-regulatory elements required for macrophage and B cell identities. Mol Cell.

[28] Love MI, Huber W, Anders S (2014). Moderated estimation of fold change and disoersion for RNA-seq data with DESeq2. Genome Biol.

[29] Karolchik D, Hinrichs AS, Furey TS, Roskin KM, Sugnet CW, Haussler D (2004). The UCSC table browser data retrieval tool. Nucleic Acids Res.

[30] Schep AN, Kummerfeld SK (2017). iheatmapr: Interactive complex heatmaps in R. JOSS.

[31] Hartley SW, Mullikin JC (2015). QoRTs: a comprehensive toolset for quality control and data processing of RNA-Seq experiments. BMC Bioinformatics.

[32] McLean CY, Bristor D, Hiller M, Clarke SL, Schaar BT, Lowe CB (2010). GREAT improves functional interpretation of cis-regulatory regions. Nat Biotechnol.

[33] Myers A, McGonigle P (2019). Overview of transgenic mouse models for Alzheimer’s disease. Curr Protoc Neurosci.

[34] Gong Z, Huang J, Xu B, Ou Z, Zhang L, Lin X (2019). Urolithin a attenuates memory impairment and neuroinflammation in APP/PS1 mice. J Neuroinflammation.

[35] Li F, Zhang Y, Lu X, Shi J, Gong Q (2019). Icariin improves the cognitive function of APP/PS1 mice via suppressing endoplasmic reticulum stress. Life Sci.

[36] Rivera-Escalera F, Pinney JJ, Owlett L, Ahmed H, Thakar J, Olschowka JA (2019). IL-1β-driven amyloid plaque clearance is associated with an expansion of transcriptionally reprogrammed microglia. J Neuroinflammation.

[37] Wang X, Sun G, Feng T, Zhang J, Huang X, Wang T (2019). Sodium oligomannate therapeutically remodels gut microbiota and suppresses gut bacterial amino acids-shaped neuroinflammation to inhibit Alzheimer’s disease progression. Cell Res.

[38] Babcock AA, Ilkjær L, Clausen BH, Villadsen B, Dissing-Olesen L, Bendixen AT (2015). Cytokine-producing microglia have an altered beta-amyloid load in aged APP/PS1 Tg mice. Brain Behav Immun.

[39] Karlic R, Chung HR, Lasserre J, Vlahovicek K, Vingron M (2010). Histone modification levels are predictive for gene expression. Proc Natl Acad Sci U S A.

[40] Heintzman ND, Stuart RK, Hon G, Fu Y, Ching CW, Hawkins RD (2007). Distinct and predictive chromatin signatures of transcriptional promoters and enhancers in the human genome. Nat Genet.

[41] Calo E, Wysocka J (2013). Modification of enhancer chromatin: what, how, and why?. Mol Cell.

[42] Dourlen P, Kilinc D, Malmanche N, Chapuis J, Lambert JC. The new genetic landscape of Alzheimer’s disease: from amyloid cascade to genetically driven synaptic failure hypothesis? Acta Neuropathol. 2019:221–36. 10.1007/s00401-019-02004-0. 10.1007/s00401-019-02004-0 PMC6660578 30982098

[43] Jung YJ, Kim YH, Bhalla M, Lee SB, Seo J. Genomics: New Light on Alzheimer’s Disease Research. Int J Mol Sci. 2018;19(12) 10.3390/ijms19123771. 10.3390/ijms19123771 PMC6321384 30486438

[44] Corces MR, Granja JM, Shams S, Louie BH, Seoane JA, Zhou W, et al. The chromatin accessibility landscape of primary human cancers. Science. 2018;362(6413) 10.1126/science.aav1898. 10.1126/science.aav1898 PMC6408149 30361341

[45] Dechassa ML, Tryndyak V, de Conti A, Xiao W, Beland FA, Pogribny IP (2018). Identification of chromatin-accessible domains in non-alcoholic steatohepatitis-derived hepatocellular carcinoma. Mol Carcinog.

[46] Ruiz JL, Tena JJ, Bancells C, Cortes A, Gomez-Skarmeta JL, Gomez-Diaz E (2018). Characterization of the accessible genome in the human malaria parasite plasmodium falciparum. Nucleic Acids Res.

[47] Qu YL, Deng CH, Luo Q, Shang XY, Wu JX, Shi Y (2019). Arid1a regulates insulin sensitivity and lipid metabolism. EBioMedicine..

[48] Buenrostro JD, Giresi PG, Zaba LC, Chang HY, Greenleaf WJ (2013). Transposition of native chromatin for multimodal regulatory analysis and personal epigenomics. Nat Methods.

[49] Sahu MR, Mondal AC. The emerging role of Hippo signaling in neurodegeneration. J Neurosci Res. 2019; 10.1002/jnr.24551. 10.1002/jnr.24551 31705587

[50] Wang Z, Lee G, Vuong R, Park JH (2019). Two-factor specification of apoptosis: TGF-β signaling acts cooperatively with ecdysone signaling to induce cell- and stage-specific apoptosis of larval neurons during metamorphosis in Drosophila melanogaster. Apoptosis..

[51] Wang SP, Wang LH. Disease implication of hyper-Hippo signaling. Open Biol. 2016; 6(10) doi: 10.1098/rsob. 160119. 10.1098/rsob.160119 PMC5090056 27805903

[52] Zetterberg H, Andreasen N, Blennow K (2004). Increased cerebrospinal fluid levels of transforming growth factor-beta1 in Alzheimer’s disease. Neurosci Lett.

[53] Rota E, Bellone G, Rocca P, Bergamasco B, Emanuelli G, Ferrero P (2006). Increased intrathecal TGF-beta1, but not IL-12, IFN-gamma and IL-10 levels in Alzheimer’s disease patients. Neurol Sci.

[54] Chong JR, Chai YL, Lee JH, Howlett D, Attems J, Ballard CG (2017). Increased transforming growth factor β2 in the neocortex of Alzheimer’s disease and dementia with Lewy bodies is correlated with disease severity and soluble Aβ42 load. J Alzheimers Dis.

[55] Salter MW, Stevens B (2017). Microgial emerge as central players in brain disease. Nat Med.

[56] Nevado-Holgado AJ, Ribe E, Thei L, Furlong L, Mayer MA, Quan J, et al. Genetic and real-world clinical data, combined with empirical validation, nominate Jak-Stat signaling as a target for Alzheimer’s disease therapeutic development. Cells. 2019;8(5) 10.3390/cells8050425. 10.3390/cells8050425 PMC6562942 31072055

[57] Boza-Serrano A, Yang Y, Paulus A, Deierborg T (2018). Innate immune alterations are elicited in microglial cells before plaque deposition in the Alzheimer’s disease mouse model 5×FAD. Sci Rep.

[58] Matsuda S, Nakagawa Y, Tsuji A, Kitagishi Y, Nakanishi A, Murai T. Implications of PI3K/AKT/PTEN signaling on superoxide dismutases expression and in the pathogenesis of Alzheimer’s disease. Diseases. 2018;6(2) 10.3390/diseases6020028. 10.3390/diseases6020028 PMC6023281 29677102

[59] McEver RP (2015). Selectins: initiators of leucocyte adhesion and signaling at the vascular wall. Cardiovasc Res.

[60] Li G, Xiong K, Korff A, Pan C, Quinn JF, Galasko DR (2015). Increased CSF E-Selectin in clinical Alzheimer’s disease without altered CSF Aβ42 and tau. J Alzheimers Dis.

[61] Haure-Mirande JV, Wang M, Audrain M, Fanutza T, Kim SH, Heja S (2019). Integrative approach to sporadic Alzheimer’s disease: deficiency of TYROBP in cerebral Aβ amyloidosis mouse normalizes clinical phenotype and complement subnetwork molecular pathology without reducing Aβ burden. Mol Psychiatry.

[62] Subramanian S, Ayala P, Wadsworth TL, Harris CJ, Vandenbark AA, Quinn JF (2010). CCR6: a biomarker for Alzheimer’s-like disease in a triple transgenic mouse model. J Alzheimers Dis.

[63] Keren-Shaul H, Spinrad A, Weiner A, Matcovitch-Natan O, Dvir-Szternfeld R, Ulland TK (2017). A unique microglia type associated with restricting development of Alzheimer’s disease. Cell..

[64] Marzi SJ, Leung SK, Ribarska T, Hannon E, Smith AR, Pishva E (2018). A histone acetylome- wide association study of Alzheimer’s disease identifies disease-associated H3K27ac differences in the entorhinal cortex. Nat Neurosci.

