# Supplementary material for: Characterization of the chromatin accessibility in an Alzheimer’s disease (AD) mouse model
Source: Alzheimers Res Ther. 2020 Mar 23;12:29. doi: 10.1186/s13195-020-00598-2 (PMC7092509; doi:10.1186/s13195-020-00598-2)
Supplement: Supplementary file 1 — Supplementary Table S1. List of primers used in ChIP-qPCR. [file 13195_2020_598_MOESM1_ESM.docx]

**Supplementary Table S1.** List of primers used in ChIP-qPCR.

| Gene name | Forward primer | Reverse primer |
| --- | --- | --- |
| *Cst7* | TGT TCT CAC TCC CAA AGC | GGA GAA CCT CAT CTT GTA |
| *Ccr6* | TGA GGA AGT GGT GTT GGG | GTC AGT AAA TCA CAG TCG |
| *Clec7a(*H3K4me3*)* | GGA TGT CCT TTT CCT CTA | GGA ATC CTC CCA CCA AAT |
| *Clec7a(*H3K27ac*)* | ATC TCA CCA TGC TGC GTT | ATC TCA CCA TGC TGC GTT |
| *Cd300lf(*H3K4me3*)* | TCA GCC TGT TGA CCA CTT | CAG CTT TTC CAG GAT GTG |
| *Cd300lf(*H3K27ac*)* | CCT GTT TAA GGG GCG ATC | CCC TAT GAA TCA GGA ACT |
| *SELE(*H3K4me3*)* | AGC AAT AAC TCA ATT CTT CC | TTA GGG CCA ATA TCA CTG |
| *SELE(*H3K27ac*)* | AGA CCA TGA GCC AGA AGT A | ATT CTT TCT GTT GCT GTC GTA |
