# Supplementary figures and images for: Characterization of the chromatin accessibility in an Alzheimer’s disease (AD) mouse model
Source: Alzheimers Res Ther. 2020 Mar 23;12:29. doi: 10.1186/s13195-020-00598-2 (PMC7092509; doi:10.1186/s13195-020-00598-2)

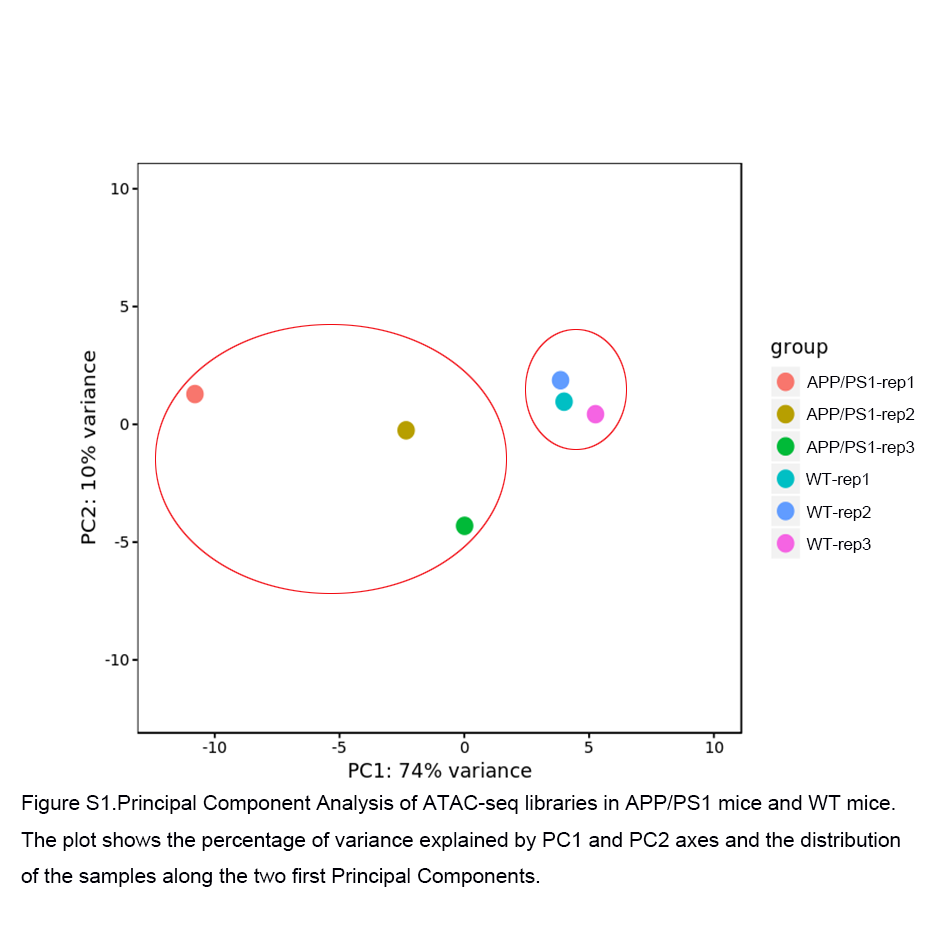

Supplement: Supplementary file 12 — Figure S1. Principal Component Analysis of ATAC-seq libraries in APP/PS1 mice and WT mice. The plot shows the percentage of variance explained by PC1 and PC2 axes and the distribution of the samples along the two first Principal Components. [file 13195_2020_598_MOESM12_ESM.tif]

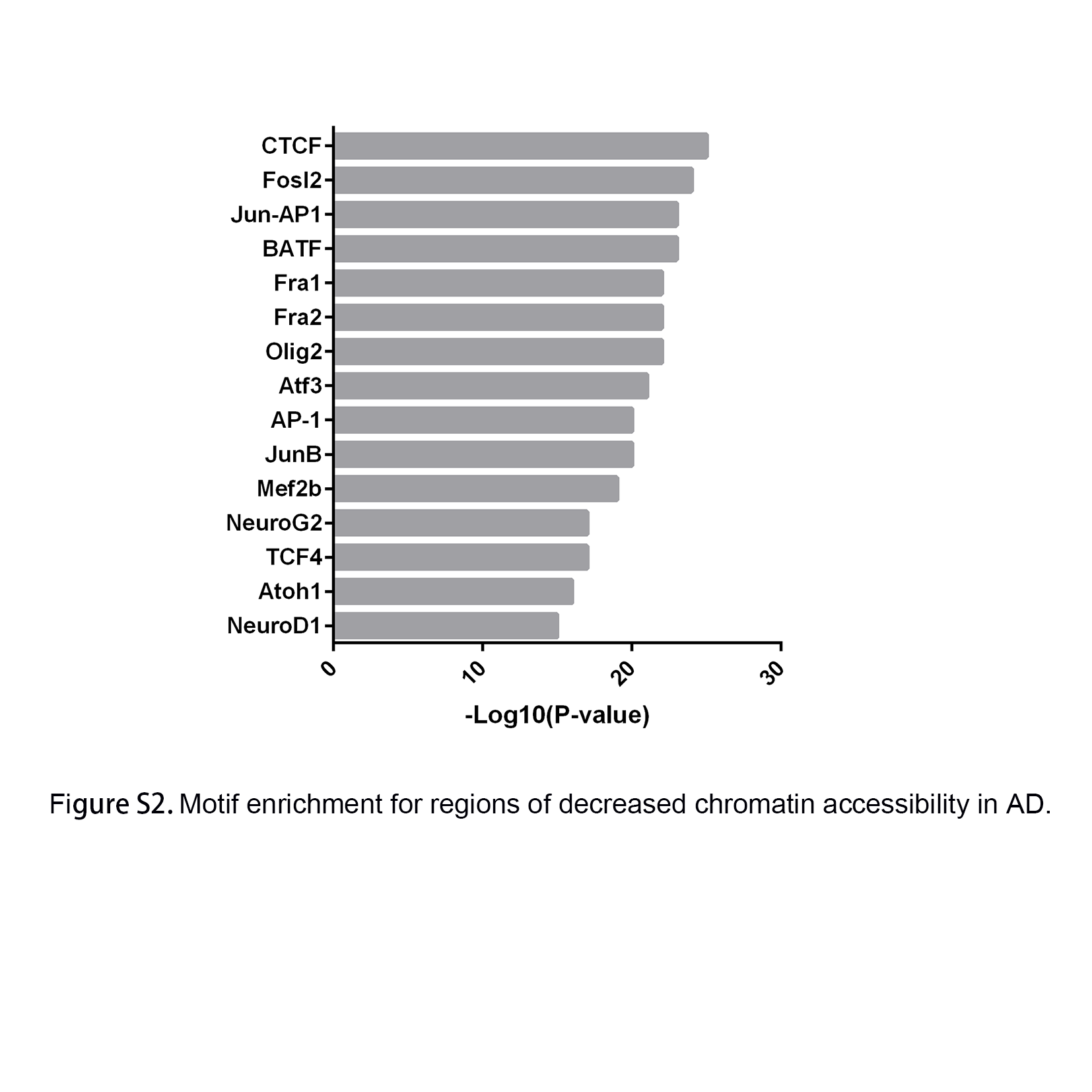

Supplement: Supplementary file 13 — Figure S2. Motif enrichment for regions of decreased chromatin accessibility in AD. [file 13195_2020_598_MOESM13_ESM.tif]
